# Supplementary material for: Human blindsight is mediated by an intact geniculo-extrastriate pathway
Source: eLife. 2015 Oct 20;4:e08935. doi: 10.7554/eLife.08935 (PMC4641435; doi:10.7554/eLife.08935)
Supplement: Supplementary file 1. — Clinical characteristics of patients. DOI: http://dx.doi.org/10.7554/eLife.08935.013 [file elife08935s001.docx]

| **Patient** | **Sex** | **Age (years)** | **Pathology** | **Time since pathology (months)** | **VFD** |
| --- | --- | --- | --- | --- | --- |
| **PB1** | M | 66 | Left occipital infarct | 8 | RHH |
| **PB2** | F | 67 | Right occipital hemorrhage | 6 | LUQ |
| **PB3** | F | 46 | Left occipital infarct | 7 | RHH |
| **PB4** | F | 38 | Right occipital tumour resection | 36 | LHH |
| **PB5** | M | 55 | Left occipital and cerebellar infarct | 18 | RHH |
| **PB6** | M | 61 | Right occipital infarct | 6 | LHH |
| **PB7** | M | 36 | Left occipital infarct | 6 | RHH |
| **PB8** | M | 70 | Left occipital infarct | 19 | RUQ |
| **PB9** | M | 60 | Left occipital infarct | 96 | RLQ |
| **PB10** | M | 30 | Left occipital infarct | 156 | RHH |
| **PB11** | F | 39 | Left occipital infarct | 7 | RHH |
| **PB12** | F | 42 | Left occipital infarct | 6 | RHH |
| **PN1** | M | 76 | Left occipital tumour resection | 21y | RHH |
| **PN2** | M | 69 | Right occipital infarct | 16 | LUQ |
| **PN3** | M | 49 | Left occipital infarct | 7y | RHH |
| **PN4** | M | 73 | Right occipital haemorrhage | 6 | LHH |
| **PN5** | M | 56 | Right occipital infarct | 36 | LHH |

**Supplementary File 1**: Clinical characteristics of patients
