# Supplementary material for: Human blindsight is mediated by an intact geniculo-extrastriate pathway
Source: eLife. 2015 Oct 20;4:e08935. doi: 10.7554/eLife.08935 (PMC4641435; doi:10.7554/eLife.08935)
Supplement: Supplementary file 2. — Number of uncleaned fascicles for the three pathways of interest in patients and control participants: (1) Ipsilateral LGN and hMT+ (2) hMT+ bilaterally via the corpus callosum (3) Ipsilateral SC and hMT+. Results are shown separately for the intact and damaged ‘ipsi-lesion’ hemispheres (right and left for control participants). DOI: http://dx.doi.org/10.7554/eLife.08935.014 [file elife08935s002.docx]

| **Subject** | **LGN <-> hMT+** | | **Crossing hMT+** | | **SC <-> hMT+** | |
| --- | --- | --- | --- | --- | --- | --- |
|  | **Ipsi-lesional** | **Contra-lesional** | **left ->** | **right ->** | **Ipsi-lesional** | **Contra-lesional** |
|  |  |  | **right** | **left** |  |  |
| **Blindsight positive patients** | | | | | | |
| **PB1** | 568 | 726 | *12* | *10* | 40 | 35 |
| **PB2** | 72 | 1470 | *11* | *9* | *5* | 78 |
| **PB3** | 348 | 490 | 352 | 360 | 35 | 37 |
| **PB4** | 55 | 2918 | *7* | *6* | *1* | 80 |
| **PB5** | 871 | 538 | 13 | 41 | 100 | 61 |
| **PB6** | 59 | 571 | 26 | 33 | *5* | 149 |
| **PB7** | 2952 | 144 | *no* | *2* | 34 | 12 |
| **PB8** | 825 | 558 | 27 | 29 | 62 | 28 |
| **PB9** | 4309 | 442 | *9* | *5* | 160 | *10* |
| **PB10** | 310 | 128 | *no* | *no* | *3* | *1* |
| **PB11** | 1716 | 186 | 37 | 34 | 18 | *9* |
| **PB12** | 1443 | 98 | 32 | 41 | 46 | *4* |
| **Blindsight negative patients** | | | | | | |
| **PN1** | 1831 | 80 | *no* | *no* | 182 | 12 |
| **PN2** | 85 | 1585 | 145 | 151 | 11 | 126 |
| **PN3** | 2928 | 590 | *8* | *6* | 33 | 108 |
| **PN4** | *no* | 741 | *no* | *no* | *no* | 123 |
| **PN5** | 25 | 1037 | *no* | *no* | *no* | 62 |
| **Controls** | | | | | | |
| **C1** | 2713 | 3174 | 159 | 148 | 88 | 69 |
| **C2** | 4406 | 2186 | *7* | *7* | 357 | 182 |
| **C3** | 559 | 369 | 17 | 28 | 112 | *10* |
| **C4** | 1640 | 1111 | 15 | 14 | 133 | 40 |
| **C5** | 778 | 189 | 17 | 14 | 113 | 30 |
| **C6** | 562 | 191 | *5* | *4* | 47 | 46 |
| **C7** | 736 | 474 | *3* | *5* | 17 | *3* |
| **C8** | 4742 | 1786 | 30 | 34 | 438 | 36 |
| **C9** | 5793 | 391 | 936 | 940 | 299 | 13 |

**Supplementary File 2.** As Table 1, but showing numbers of uncleaned tracts.
